# Sperm DNA integrity in adult survivors of paediatric leukemia and lymphoma: A pilot study on the impact of age and type of treatment

**DOI:** 10.1371/journal.pone.0226262

**Published:** 2019-12-19

**Authors:** Hermance Beaud, Océane Albert, Bernard Robaire, Marie Claude Rousseau, Peter T. K. Chan, Geraldine Delbes

**Affiliations:** 1 INRS-Centre Armand-Frappier Santé Biotechnologie, Laval, Quebec, Canada; 2 Department of Pharmacology and Therapeutics, McGill University, Montreal, Quebec Canada; 3 Department of Obstetrics & Gynecology, McGill University, Montreal, Quebec, Canada; 4 Division of Urology, McGill University Health Center, Montreal, Quebec, Canada; Universite Clermont Auvergne, FRANCE

## Abstract

Childhood cancer survivors (CCS) are more likely than siblings to report low sperm count and to use assisted reproductive technologies. Yet, it is still unclear if the sperm produced many years after remission of cancer display DNA and chromatin damage linked to male infertility and poor embryo development. As well, the importance of the age at diagnosis in relation to puberty is poorly understood. In this pilot study, we compared reproductive parameters and sperm damage from adult survivors of childhood leukemia and lymphoma, sub-divided into those diagnosed before or after puberty, to men with no history of cancer. Our data indicate that CCS, independently of the age of diagnosis, have a high risk of low sperm count and when sperm are present, chances of DNA and chromatin abnormalities appear similar to those seen in the general population. Exposure to alkylating agents is correlated with low sperm count whereas exposure to anthracyclines, and doxorubicin in particular, could have long-term consequences on sperm integrity. This study highlights the need for further research on fertility among male CCS and the importance of informing families about the potential long-term impact of chemotherapy on male fertility regardless of age at diagnosis.

## Introduction

The incidence of childhood cancer has been steadily increasing worldwide over the past 50 years [1–4]. The success of treatments and consequent higher survival after childhood cancer has led to a shift in parental and patients’ worries. While survival was the primary concern, now, the late effects of cancer and its treatment are becoming major healthcare issues [5–7]. Epidemiological studies from more than 11,000 male participants in the *Childhood Cancer Survivor Study* have shown a significant decrease in male fertility when compared to their healthy siblings [5, 8, 9]. In particular, those with a diagnosis of Hodgkin lymphoma (HL), non-Hodgkin lymphoma (NHL) or leukemia were less likely to get their partners pregnant (hazard ratio 0.34, 0.60 and 0.70 respectively) [5].

The most common cause of infertility in these patients is low sperm count or azoospermia [10]. Studies on sperm from adult cancer patients showed that cancer itself and its treatment induced sperm aneuploidy, chromatin damage, and epigenetic changes that persist years post-chemotherapy [11–15]. In the case of childhood cancer, however, it is still unclear whether the sperm produced years after remission of cancer have chromatin and DNA damage. Large epidemiological studies have shown lower birth rates among male cancer survivors and a slightly higher risk of major congenital abnormalities among their offspring (reviewed in [16]). Such defects may be due to the potential mutagenic effects of anti-cancer drugs on the paternal germline. To date, only two studies assessed sperm chromatin integrity in CCS and observed low risk of sperm damage compared to age-matched controls [17, 18]. But, because such cohorts are very hard to recruit, both studies grouped CCS with various diagnostics and heterogeneous treatments making it difficult to correlate the impact of one drug to sperm outcome. As well, they used CCS with a large range of age at diagnosis, so analyses were done without segregating the impact of pre-pubertal and post-pubertal treatment. While it was thought that being pre-pubertal during anti-cancer therapy conferred protection against gonadal damage, evidence of the impact on long-term sperm production has led some researchers to conclude that the prepubertal gonad is even more vulnerable to the cytotoxic effects of chemotherapy than the adult testis [19]. Yet, the importance of age at diagnosis in relation to puberty, on potential long-term effect such as sperm production or sperm DNA and chromatin quality remains poorly understood.

In this pilot study, we aimed to specifically address issues of the influence of age at diagnosis or the type of treatment received on sperm count, sperm chromatin and sperm DNA integrity in men with a history of childhood cancer. To this end, we recruited subjects with specific types of cancer and treatments along with well-defined ages of diagnosis.

## Results

### 1- Characteristics of participants

In order to limit variations in types of cancer and treatment, we have targeted our recruitment to adult survivors of three of the most prevalent paediatric hematologic malignancies, namely acute lymphocytic leukemia (ALL), HL and NHL. Reproductive parameters of these CCS are compared to those of age-matched men who have never been diagnosed with cancer. To distinguish impacts on immature or mature testis, the CCS group was subdivided according to the age at diagnosis relative to the onset of puberty as defined by the US and European consortium [20]: diagnosed before puberty (ages 4–14, n = 6) and diagnosed after puberty (ages 15–17, n = 7) (Table 1). At the time of recruitment, the age of participants was similar across groups, but time in remission was significantly longer for those diagnosed before puberty compared to those diagnosed after puberty (Table 1). Interestingly, we observed that CCS had a higher body mass index than controls, although this was significant only for CCS diagnosed after puberty (Table 1).

**Table 1:** Personal and medical characteristics of participants.

Characteristics	Controls	CCS	CCS diagnosed	CCS diagnosed
			before puberty	after puberty
	(n = 12)	(n = 13)	(n = 6)	(n = 7)
Diagnosis	**-**	3 ALL	2 ALL	1 ALL
		8 HL	2 HL	6 HL
		2 NHL	2 NHL	
Age at diagnosis	**-**	12.8 ± 1.3	9.2 ± 1.8	16.0 ± 0.4^a^
(years, mean ± SEM)				
Age at recruitment	30.7 ± 1.7	27.8 ± 1.6	30.3 ± 2.1	25.6 ± 2.2
(years, mean ± SEM)				
Remission	**-**	13.4 ± 2.3	19.6 ± 3.9	9.0 ± 2.2^a^
(years, mean ± SEM)				
BMI	22.9 ± 0.5	26.3 ± 1.2^b^	24.5 ± 0.7	27.8 ± 2.0^b^
(kg/m^2^, mean ± SEM)				
University graduate	66.7 (39.1–86.2)	46.2 (23.2–70.9)	50.0 (18.8–81.2)	42.9 (15.8–75.0)
(%, 95% CI)				
Smoker at interview	16.7 (4.7–44.8)	23.1 (8.2–50.3)	16.7 (3.0–56.4)	28.6 (8.2–64.1)
(%, 95% CI)				
>5 alcoholic drinks/week	33.3 (13.8–60.9)	30.8 (12.7–57.6)	33.3 (9.7–70.0)	28.6 (8.2–64.1)

CCS: male Childhood Cancer Survivor, ALL: Acute Lymphoblastic Leukemia, HL: Hodgkin's Lymphoma, NHL: Non-Hodgkin's Lymphoma, CI: confidence interval.

^a^: p<0.01 using an unpaired Student's t-test compared to CCS diagnosed before puberty.

^b^: p<0.01 using a Mann Whitney test compared to controls.

### 2- Reproductive parameters

**Fig 1:**
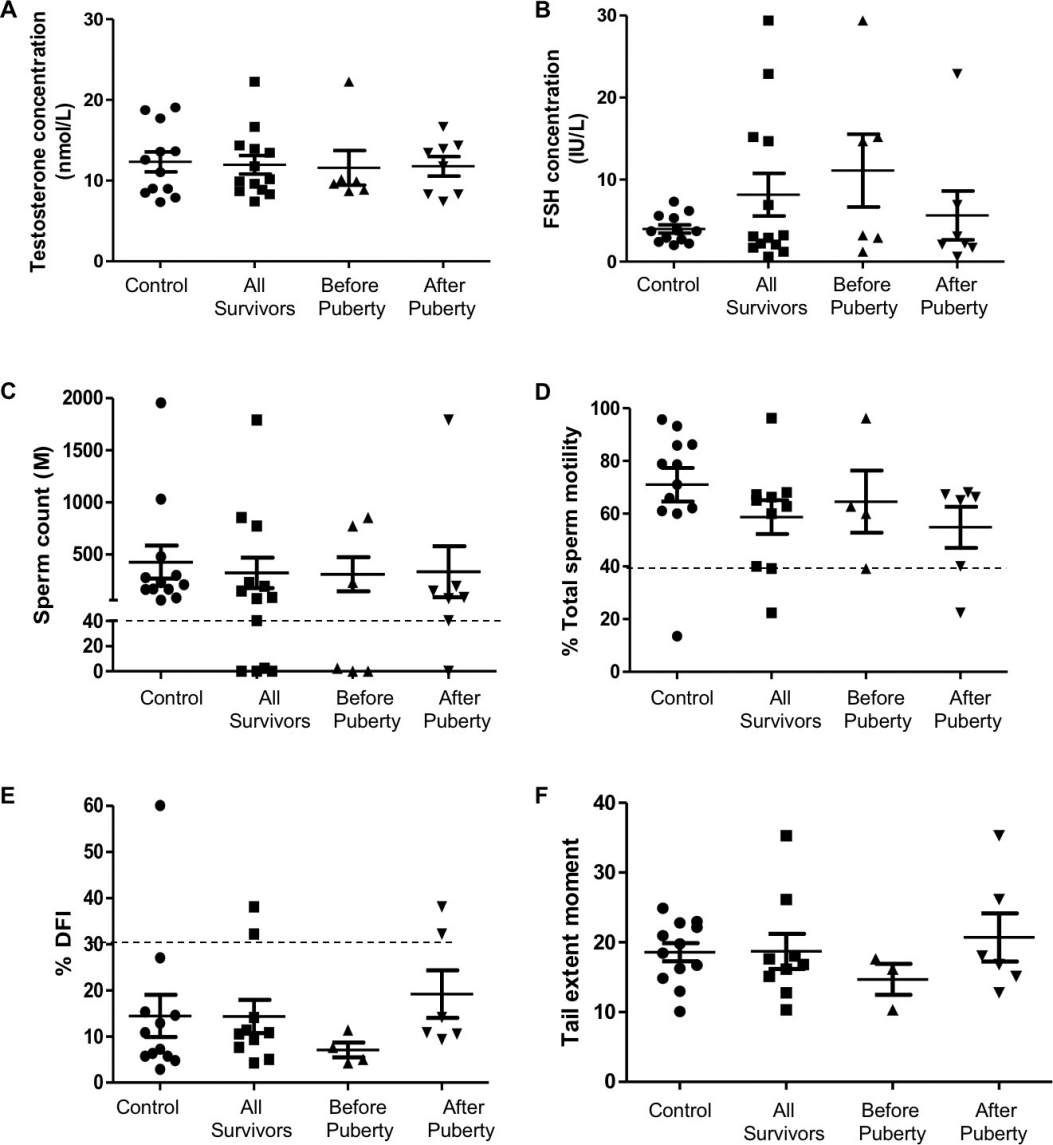
Reproductive parameters in adult controls and CCS. Circulating levels of (A) Total testosterone and (B) Follicle-Stimulating Hormone (FSH) have been measured in serum from participants using immuno-enzymatic chemiluminescent assays. Semen parameters are illustrated by (C) sperm count and (D) sperm total motility measured by CASA for each participants. Sperm chromatin and DNA integrity have been studied using the (E) sperm DNA fragmentation index (DFI) and (F) the percentage of DNA in the COMET tail. All data are expressed as mean ± SEM and each point represents an individual value. Dashed lines represent threshold values in humans according to (WHO and Evenson, 2016). Comparisons of average values between groups were done using a Student’s t-test or a Mann-Whitney test according to the normality test results.

No differences were observed in the mean serum testosterone and FSH between controls and CCS and, further, between the controls and the two subgroups of CCS (Fig 1A and 1B). Individual values revealed that all participants have values within the normal ranges, except for 4 CCS who have FSH levels higher than the clinical standard of 8IUs/L that suggests non obstructive azoo/oligozoospermia (Fig 1B). Importantly, these elevated FSH values were observed in CCS regardless of the age at diagnosis.

Standard semen analysis showed that our control group displayed normal sperm parameters according to the World Health Organization (WHO) standards [21] except for one individual who had a low percentage of total sperm motility (Fig 1C and 1D and S1 Table). No difference in average sperm count or motility was observed across groups (Fig 1C and 1D). Because of the small sample size, the statistical power to observe differences in average values is low, but the prevalence of about 38% with low sperm count that we observed corresponds to what has been described by others for childhood cancer survivors [18, 22–25]. Moreover, individual values revealed that 5 out of the 13 CCS had sperm concentrations below the WHO standards of 15 million /ml (Fig 1C); 3 samples were azoospermic and 2 were oligozoospermic (10.9 and 1.4 million sperm/ml). Interestingly, azoo/oligozoospermia was observed regardless of the age at diagnosis as survivors diagnosed before and after puberty were both affected.

Analysis of sperm chromatin and DNA integrity revealed no difference between the average values of all groups (Fig 1E and 1F). Interestingly, individual values revealed that two controls and two CCS diagnosed after puberty had DNA Fragmentation Index (DFI) higher than the threshold established at 25% [26] (Fig 1E). Despite the absence of a consensus threshold value for the COMET assay, we observed that both CCS previously described with high %DFI displayed the highest values for the COMET parameters.

### 3-Correlations between sperm parameters and treatment characteristics

Despite the limited number of subjects, our pilot study allows to reveal the expected strong positive correlation between sperm count and sperm motility (Table 2). As well, the fact that sperm count did not correlate with sperm quality parameters such as the % DFI and the %Tail DNA confirmed that these assays target different parameters (Table 2). In order to determine if the azoo-, oligo- or asthenozoospermia, high %DFI or high tail DNA observed in CCS were associated with the type of treatment received, these variables were correlated with treatment characteristics based on the participants’ medical record. Treatments and doses received are summarized in S2 Table according to the 3 main classes of drugs received: vinca alkaloids, alkylating agents and anthracyclines. We observed a significant negative correlation between sperm count and the cumulative dose of alkylating agent, determined by the cyclophosphamide equivalent dose (CED) [25] (Table 2). No other drugs correlated negativelly with sperm count (Table 2). Interestingly, we observed a strong positive correlation between the cumulative dose of anthracyclines and the %DFI (Table 2). Our study did not reveal a link between any sperm parameters and the duration of treatment, the time of remission or the age at diagnosis (Table 2).

**Table 2:** Correlations between sperm parameters and cancer treatment characteristics.

	Sperm count	Sperm motility	%DFI	%Tail DNA
**Sperm count**		0.85*	0.19	-0.07
**%DFI**	0.19	-0.31		
**%Tail DNA**	-0.07	-0.67	0.53	
**Vinca Alkaloids**^**b**^	0.07	-0.16	0.16	-0.21
**Alkylating agents (CED**^**a**^**)**	-0.62*	-0.52	0.31	0.00
**Anthracyclines**^**b**^	-0.02	-0.47	0.92*	0.71
**Duration of treatment**	0.13	0.01	-0.17	0.25
**Time of remission**	-0.40	-0.33	-0.25	0.00
**Age at diagnosis**	0.13	0.09	0.47	-0.18

Correlation coefficients determined by the Spearman test are shown.

* p≤0.05 using the Spearman test.

^a^: CED = 1.0 (cumulative cyclophosphamide dose (mg/m^2^)) + 0.857 (cumulative procarbazine dose (mg/m^2^)) + 100 (cumulative nitrogen mustard dose (mg/m^2^)).

^**b**^:The cumulative doses of vinca alkaloids or anthracyclines were determined by adding cumulative doses of each compounds received.

## Conclusion / Discussion

Overall, our results are in line with other studies and reinforce the fact that CCS have higher prevalence of no or low sperm count. Importantly, both groups of CCS had comparable prevalence of azoo/oligozoospermia, supporting the premise that the prepubertal testis is not protected from injury after a history of pediatric cancer [19]. Azoo/oligozoospermia was correlated with the cumulative dose of alkylating agents received, but the possibility of infertility could not be predicted based on the age of diagnosis and treatment. As fifty percent of men diagnosed in 2000 to 2004 have no memory of counselling on the risk of infertility following childhood cancer treatment [27], our study underlines the importance of informing families about the potential long-term impact on male fertility before anti-cancer treatment starts, and this recommendation should be considered regardless of age at diagnosis. In parallel, we did not measure a difference in the incidence of sperm chromatin or DNA damage in CCS when compared to controls. However, by considering individual values and age at diagnosis, we observed that the two CCS displaying %DFI above 25% and the highest %Tail DNA were diagnosed after puberty. Importantly, larger studies are greatly needed to further investigate if the maturity of the testis modulates the sensitivity to the treatments and the long-term impact of sperm quality. As well, our data suggest that sperm DNA integrity was correlated to cumulative dose of anthracyclines. The suggestion that anthracyclines could induce long-term impact on sperm DNA integrity is supported by experimental data on pre-pubertal rats [28] and *in vitro* studies [29, 30]. Yet larger studies, providing greater statistical power and allowing to adjust for the effect of several characteristics of the participants simultaneously, will be needed to determine if these associations can be replicated. Also, mechanistic studies on how chemotherapeutic compounds can have long-term effects on sperm quality are greatly needed to help reduce the side effects of cancer treatment and improve the quality of life of CCS.

## Methods

### 1. Recruitment of participants

Adult participants were recruited from follow-up oncology clinics at the McGill University Health Center (Montreal, Canada) and from the general public with compensation for transportation fees. This study was approved by the Institutional Ethics Review Boards from the Faculty of Medicine at McGill University (IRB #A01-M03-14A) and INRS (CER 15–376). Informed and written consents were obtained from each participant recruited. Inclusion criteria were: 1) be over 18 years old to consent to participate; 2) be below the age of 40 years; 3) have completed treatment before the age of 19 years; 4) be in remission for at least 3 years with respect to their cancer; 5) be able to ejaculate by masturbation to provide semen samples; 6) be free of additional co-morbidities that can affect their fertility status as evaluated by an eligibility questionnaire (e.g., cryptorchidism, varicocele, testicular cancer, vasectomy). Exclusion criteria were: 1) inability to provide informed consent; 2) received total body or pelvic radiation treatment. Each participant completed a questionnaire which elicited information on personal characteristics and lifestyle such as education level, height, weight, smoking, and alcohol consumption status. Written consent from each participant was obtained to allow access to their medical records. Details on the diagnosis, the age at diagnosis as well as type of cancer, and the doses and duration of treatment were collected retrospectively when available (Table 1 and S2 Table).

### 2. Samples collection, preparation and analysis

From each participant, serum samples were obtained and assayed for total testosterone, estradiol (E2), luteinizing hormone (LH), follicle-stimulating hormone (FSH), albumin, and sex hormone-binding globulin (SHBG) using immuno-enzymatic chemiluminescent assays (Beckman Coulter Inc., Brea, CA) as per the manufacturer's instructions. A semen sample was collected after 3 to 7 days of abstinence by masturbation. Semen analysis were done on semen sample collected by masturbation after 3 to 7 days of abstinence, within one hour after collection, by CASA (SpermVision; Sperm Processor Pvt. Ltd, Garkheda, India) [31]. When sperm was lower than 5M/ml, samples were centrifuged and concentration was further confirmed using a hemocytometer [21]. As previously described, semen samples were aliquoted and stored at -80°C in the absence of cryoprotectants before sperm chromatin analysis [32]. The maximum storage time was 4 years. Sperm chromatin and DNA integrity were evaluated amongst non-azoospermic subjects by the sperm chromatin structure assay and the COMET assay respectively, as previously described (Albert, et al., 2016)

## Supporting information

S1 Table Additional semen parameters in adult controls and CCS. (DOCX)

S2 Table Diagnosis and sperm concentration in relation to cancer treatment. Details on the molecules and the cumulative dose received are given for each participating CCS according to the classes of drugs, Underlined numbers correspond to male CCS diagnosed before puberty.
(DOCX)
