# Supplementary material for: Sperm DNA integrity in adult survivors of paediatric leukemia and lymphoma: A pilot study on the impact of age and type of treatment
Source: PLoS One. 2019 Dec 19;14(12):e0226262. doi: 10.1371/journal.pone.0226262 (PMC6922400; doi:10.1371/journal.pone.0226262)
Supplement: S1 Table — (DOCX) [file pone.0226262.s001.docx]

**S1 Table : Additional semen parameters in adult controls and CCS.**

| **Characteristics** | **Controls**  **(n=12)** | **CCS**  **(n=9)** | **CCS diagnosed before puberty**  **(n=4)** | **CCS diagnosed after puberty**  **(n=5)** |
| --- | --- | --- | --- | --- |
| Rapid progressive (%) | 34.81 ± 4.63 | 30.52 ± 6.64 | 35.17 ± 9.48 | 26.81 ± 9.86 |
| Slow progressive (%) | 20.95 ± 3.28 | 15.12 ± 2.88 | 17.09 ± 4.74 | 13.54 ± 3.86 |
| VAP (μm/sec) | 44.54 ± 2.4 | 38.49 ± 3.30 | 40.62 ± 5.34 | 36.79 ± 4.51 |
| VCL (μm/sec) | 66.80 ± 3.89 | 57.40 ± 6.27 | 58.53 ± 9.71 | 56.50 ± 9.18 |
| VSL (μm/sec) | 33.35 ± 1.99 | 30.19 ± 2.72 | 33.24 ± 3.89 | 27.75 ± 3.77 |
| STR | 0.74 ± 0.02 | 0.78 ± 0.02 | 0.82 ±0.04 | 0.75 ± 0.02 |
| LIN | 0.5 ± 0.02 | 0.54 ± 0.03 | 0.59 ± 0.06 | 0.50 ± 0.03 |
| ALH (μm) | 2.12 ± 0.11 | 2.02 ± 0.21 | 2.17 ± 0.41 | 1.89 ± 0.22 |
| BCF (Hertz) | 25.38 ± 0.82 | 25.92 ± 0.55 | 25.91 ± 0.23 | 25.93 ± 1.04 |

# CCS: male Childhood Cancer Survivor, Sperm parameters were measured by CASA for each participant. Data are expressed as mean ± SEM.  VAP = average path velocity; VCL = curvilinear velocity; VSL = straight line velocity; STR = straightness; LIN = linearity; ALH = amplitude of lateral head movement; BCF = beat cross frequency
