# Supplementary material for: Sperm DNA integrity in adult survivors of paediatric leukemia and lymphoma: A pilot study on the impact of age and type of treatment
Source: PLoS One. 2019 Dec 19;14(12):e0226262. doi: 10.1371/journal.pone.0226262 (PMC6922400; doi:10.1371/journal.pone.0226262)
Supplement: S2 Table — Details on the molecules and the cumulative dose received are given for each participating CCS according to the classes of drugs, Underlined numbers correspond to male CCS diagnosed before puberty. (DOCX) [file pone.0226262.s002.docx]

**S2 Table : Diagnosis and sperm concentration in relation to cancer treatment**. Details on the molecules and the cumulative dose received are given for each participating CCS according to the classes of drugs, Underlined numbers correspond to male CCS diagnosed before puberty.

| **CCS** | **Type** | **Sperm** | **Vinca** | **Alkylating** | **Anthracyclines** | **Corticosteroids** | **Other** |
| --- | --- | --- | --- | --- | --- | --- | --- |
|  | **of** | **count** | **Alkaloids** | **agents** | **(mg/m^2^)** | **(mg/m^2^)** |  |
|  | **cancer** | **(Millions)** | **(mg/m^2^)** | **(CED mg/m^2^)** |  |  |  |
| **#1** | HL | 0 | Vincristine | Mustargen | DXO (200) | Prednisone (40) | - |
|  |  |  | Vinblastine (60) | Procarbazine (4286) | Bleomycin (80) | Dexamethasone |  |
| **#2** | NHL | 2.4 | Vincristine (11) | CPA (2250) | DXO (120) | Prednisone (360) | G-CSF |
|  |  |  |  |  |  | Hydrocortisone | Methotrexate |
|  |  |  |  |  |  |  | Cytarabine |
| **#3** | ALL | 854.7 | Vincristine (30) | - | - | Prednisone (4440) | Methotrexate |
|  |  |  |  |  |  |  | L-Asp. |
|  |  |  |  |  |  |  | Cytarabine |
|  |  |  |  |  |  |  | 6-MP |
| **#4** | HL | 0 | Vincristine | Mustargen (4800) | DXO (175) | Dexamethasone | Radiation |
|  |  |  | Vinblastine (54) |  | Bleomycin (70) |  |  |
| **#5** | HL | 40.1 | Vincristine (7) | CPA (5250) | DXO (350) | Prednisone (188) | Radiation |
|  |  |  |  |  |  | Dexamethasone | Rituxan |
| **#6** | HL | 76.3 | Vincristine (8) | CPA (3600) | DXO (150) | Prednisone (840) | Radiation |
|  |  |  |  |  |  |  | G-CSF |
| **#7** | HL | 88.2 | Vincristine (44.8) | CPA (3200) | DXO (200) | Prednisone (1120) | Radiation |
|  |  |  |  |  | Bleomycin (60) |  | Etoposide |
|  |  |  |  |  |  |  | G-CSF |
| **#8** | HL | 147.4 | Vincristine (11.2) | CPA (5200) | DXO (100) | Prednisone (1120) | Radiation |
|  |  |  |  |  | Bleomycin (60) | Hydrocortisone | Etoposide |
|  |  |  |  |  |  |  | G-CSF |
|  |  |  |  |  |  |  | Singulair |
| **#9** | HL | 194.6 | Vincristine (10.5) | - | DXO (175) | - | Etoposide |
|  |  |  |  |  | Bleomycin (70) |  |  |
| **#10** | ALL | 1792.3 | Vincristine (78) | - | DXO (360) | Prednisone (20040) | Radiation |
|  |  |  |  |  |  | Hydrocortisone | Septra |
|  |  |  |  |  |  |  | Methotrexate |
|  |  |  |  |  |  |  | L-Asp. |
|  |  |  |  |  |  |  | Cytarabine |
|  |  |  |  |  |  |  | 6-MP |

CCS: Childhood Cancer Survivor, ALL: Acute Lymphoblastic Leukemia, HL: Hodgkin's Lymphoma, NHL: Non-Hodgkin's Lymphoma, CPA: Cyclophosphamide, DXO: Doxorubicin, L-Asp: L-Asparaginase, 6-MP: mercaptopurine.
